# Supplementary material for: To Kill or to Repel Mosquitoes? Exploring Two Strategies for Protecting Humans and Reducing Vector-Borne Disease Risks by Using Pyrethroids as Spatial Repellents
Source: Pathogens. 2021 Sep 11;10(9):1171. doi: 10.3390/pathogens10091171 (PMC8471886; doi:10.3390/pathogens10091171)
Supplement: Supplementary file 1 [file pathogens-10-01171-s001.zip › pathogens-1348671-supplementary.pdf]

pathogens MDPI

Supplementary materials

Table S1. Parameters used in the Scenario 1 ConsExpo model.

Parameter		Value
**AS concentration (% [w/w])**		0.104
Potential body exposure (mg/min)[1]		45.20
Potential hand exposure (mg/min)[1]		64.7
Potential inhalation exposure (mg/m³)[1]		35.9
Inhalation/respiration rate (m^3/h)[2]		1.25
Application duration (s)[3]		9
Dermal absorption (%)[4]	TFT	50
Body mass (kg)[2]		60

[1]Default BHHEM value [38]

[2]Recommendation no. 14 of the Biocidal Products Committee (BPC) Ad hoc Working Group on Human Exposure—default values when assessing human exposure to biocidal products [40]

[3]Recommended spray duration based on mode of use

[4]Default value based on EFSA guidance on dermal absorption [82]

Table S2. Parameters used in the Scenario 2 ConsExpo model.

Parameters		Value
**AS concentration (% [w/w])**		0.104
Vapour pressure (kPa)	TFT	9×10^{-7}
Molar mass (g/mol)	TFT	371
Parameters in the exposure to vapour: evaporation model	Product amount (g)[1]	12.3
	Emission duration (h)[1]	24
	Exposure duration (min)[1]	720
	Room volume (m^3)[2]	30
	Ventilation rate (1/h)[2]	0.5
	Mass transfer coefficient (m/hr)[3]	10
	Release area (m^2)[2]	12
Body mass (kg)[4]	Adult	60
	Toddler	10

[1]Product-specific information

[2]Pest Control Products Fact Sheet [39]

[3]Cleaning Products Fact Sheet—2018 update [83]

[4]Recommendation no. 14 of the BPC Ad hoc Working Group on Human Exposure—default values when assessing human exposure to biocidal products [40]

Table S3. Parameters used in the Scenario 3 ConsExpo model.

**Parameters**		Value
**AS concentration (% [w/w])**		0.104
Dislodgeable residues[1] (mg/cm²)	Outdoors	0.0574
Skin surface area (cm²)[2]	Adult	9,550
	Toddler	2,419.2
Surface contamination (%)		100
Dermal absorption (%)[3]		70
Body mass (kg)[4]	Adult	60
	Toddler	10

[1]Default values from ECHA Technical Notes for Guidance Part 2 (p. 257; in cases of non-professional use and residential exposure to biocides) [84]

[2]Based on recommendation no. 14 of the BPC Ad hoc Working Group on Human Exposure [40], where skin surface areas were calculated as follows:

adults: 820 cm^2 for hands + 2,270 cm^2 for arms + 1,130 cm^2 for feet + 5,330 cm^2 for legs = 9,550 cm^2

toddlers: 230.4 cm^2 for hands + 681.6 cm^2 for arms + 288 cm^2 for feet + 1,219.2 cm^2 for legs = 2,419.2 cm^2

[3]Default value from EFSA guidance on dermal absorption [82]

[4]Recommendation no. 14 of the BPC Ad hoc Working Group on Human Exposure—default values when assessing human exposure to biocidal products [40]

Table S4. Parameters used in the Scenario 4 ConsExpo model.

**Parameters**		Value
**AS concentration (% [w/w])**		0.104
Dislodgeable residues[1] (mg/cm²)	Outdoors	0.0574
Surface area of fingertips (cm²)[2]		4
Efficiency of removal by saliva from skin (%)[3]		50
Number of hand-to-mouth contacts (per day)[3]		36
Oral absorption (%)[4]		100
Body mass (kg)[5]	Toddler	10

[1]Default values from ECHA Technical Notes for Guidance Part 2 (p. 257; in cases of non-professional use and residential exposure to biocides) [84]

[2]According to the Do-It-Yourself Products Fact Sheet (RIVM report 320104007/2007; assuming a fingertip surface area of 1 cm^2) and utilising four fingers as a worst-case situation [85]

[3]Camann DE, Majumadar TK, and Geno P, 2000. Evaluation of Saliva and Artificial Salivary Fluids for Removal of Pesticide Residues from Human Skin. Final Report to EPA by ManTech (Contract 68-D5-0049) [86]

[4]TFT Assessment Report NL (2014) [44]

[5]Recommendation no. 14 of the BPC Ad hoc Working Group on Human Exposure—default values when assessing human exposure to biocidal products [40]
